# The Tibial Sag Radiograph Is a Reliable Radiographic Technique to Quantify Posterior Cruciate Ligament Laxity

**DOI:** 10.1002/ars2.70142

**Published:** 2026-10-26

**Authors:** Geoffrey W. Schemitsch, Darius L. Lameire, Caroline Cristofaro, Graeme Hoit, Daniel B. Whelan

**Affiliations:** ^1^ Department of Surgery, Division of Orthopaedic Surgery University of Toronto Toronto Ontario Canada; ^2^ Institute of Health Policy, Management and Evaluation University of Toronto Toronto Ontario Canada; ^3^ Division of Orthopaedic Surgery St. Michael's Hospital Toronto Ontario Canada

## Abstract

**Purpose:**

To evaluate the interobserver and intraobserver reliability associated with using tibial sag stress radiographs as a measure of posterior laxity in patients with posterior cruciate ligament injuries.

**Methods:**

All patients with posterior cruciate ligament injuries who underwent stress radiographic measurements using bilateral tibial sag stress radiographs and Telos stress radiographs between December 2018 and July 2024 were identified from an institutional database. All radiographs were measured by 3 independent blinded observers. The magnitude of posterior laxity was calculated by taking the side‐to‐side difference (SSD) in posterior tibial sag between the injured and uninjured limb. Clinical laxity of injured knees was assessed by a fellowship‐trained sports surgeon. Intraclass correlation coefficients (ICCs) were calculated to assess the interobserver and intraobserver reliability of each stress radiograph technique.

**Results:**

Among the 74 included knees, there were 26 (35.1%) grade I injuries, 39 (52.7%) grade II injuries, and 9 (12.2%) grade III injuries. The mean age of the cohort was 34.4 ± 12.1 years (range, 14‐67 years), and 54 (73%) were men. The tibial sag method showed good to excellent interobserver reliability (ICC 0.89, 95% confidence interval [95% CI] 0.84‐0.92) in measuring SSD. The Telos method showed moderate to good interobserver reliability (ICC 0.70, 95% CI 0.59‐0.78). The intraobserver reliability of measuring SSD using the tibial sag method was excellent for 2 reviewers (ICC 0.96, 95% CI 0.94‐0.97; ICC 0.95, 95% CI 0.92‐0.97) and good to excellent for 1 reviewer (ICC 0.91, 95% CI 0.86‐0.94).

**Conclusions:**

Tibial sag radiography is a reliable method that can be used to identify posterior displacement of the tibia relative to the femur as measured by SSD in patients with posterior cruciate ligament injuries.

**Level of Evidence:**

Level III, diagnostic retrospective comparative study.

## Graphical abstract

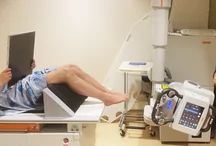

Stress radiographs provide objective evidence of knee laxity in the evaluation of ligamentous knee injuries.^1^, ^2^ Comparing side‐to‐side differences (SSDs) in relative knee joint laxity, between the injured and uninjured knee, can help distinguish pathologic laxity as a result of injury from global hypermobility and aid in evaluating the severity of ligamentous injuries.^2^, ^3^ Postoperative evaluation may also represent an important application of stress radiography in clinical research.^1^

Several stress radiography techniques have been proposed to measure posterior laxity in the posterior cruciate ligament (PCL) deficient knee.^3^, ^4^, ^5^ Clinical examination of posterior laxity with posterior drawer testing is commonly performed with the knee flexed at 90°.^6^, ^7^ Therefore, most posterior stress radiograph techniques capture a lateral knee radiograph while a posterior directed force (Telos device, kneeling, and hamstring contraction) is applied to the anterior aspect of the tibia with the knee flexed at 90°.^8^ These techniques can be challenging to administer in the setting of an acutely injured knee, which often cannot be flexed sufficiently, and in those with anterior knee pain or injuries that prohibit the application of an external force to the anterior tibia.^8^, ^9^

Skyline view knee radiographs have been described in various forms.^10^, ^11^, ^12^ Although these views are primarily used to assess disease of the patellofemoral joint, a modified method has been described to evaluate PCL injuries.^11^ This uses a “coned out” skyline view of the bilateral patellofemoral joints with the knees positioned at approximately 70° of flexion and increased brightness to allow for identification of the position of the anterior tibial plateau relative to the distal femur. In the presence of a PCL injury, the injured knee shows a relative tibial sag when compared with the uninjured side, in response to gravity stress.

We describe the technique to acquire and measure tibial sag stress radiographs to assess PCL deficiency. The purpose of this study was to evaluate the interobserver reliability and intraobserver reliability associated with using tibial sag stress radiographs as a measure of posterior laxity in patients with PCL injuries. We hypothesized that the tibial sag method would show at least good reliability.

## METHODS

We conducted a retrospective review of a prospectively collected institutional database capturing knee ligamentous injuries. The study protocol received Institutional Review Board approval (Unity Health Toronto Research Ethics #12‐027). Inclusion criteria were magnetic imaging resonance confirmed PCL injury, availability of tibial sag and Telos stress radiographs, and treatment at 1 institution by a single surgeon (D.B.W.) between December 2018 and July 2024. Patients who were unable to flex their knee to 90° were not captured in this study as they could not undergo Telos stress radiographs. Clinical laxity was subjectively graded (I, II, or III) by assessing the degree of posterior sag on posterior drawer testing.^13^ Laxity was classified as grade I when the tibial plateau remained anterior to the femoral condyle; grade II when the tibial plateau was flush with the femoral condyle; and grade III when the tibial plateau translated posterior to the femoral condyle.^13^ All posterior drawer tests were performed by a single sports fellowship‐trained orthopaedic surgeon (D.B.W.) with >20 years of clinical experience in knee ligament reconstruction.

### Tibial Sag Radiograph Technique

One radiographic technician and one surgeon (D.B.W.) administered all stress radiographs. The patient was positioned supine with both knees flexed at 70°. A radiolucent triangular bump was positioned under the distal femur, one hand's breath from the knee joint, allowing the tibia to sag relative to the femur (Bone Foam, Corcoran, MN; Figures 1 and 2). The ankles were rested on the edge of the radiograph table to ensure no axial pressure was applied to the knee. Rotation of the knee was standardized by ensuring the patellae were positioned perpendicular to the radiograph table. The patient was encouraged to relax all muscles crossing the knee joint while maintaining this center positioning of their knees (through hip activation, not knee activation).

**FIGURE 1:**
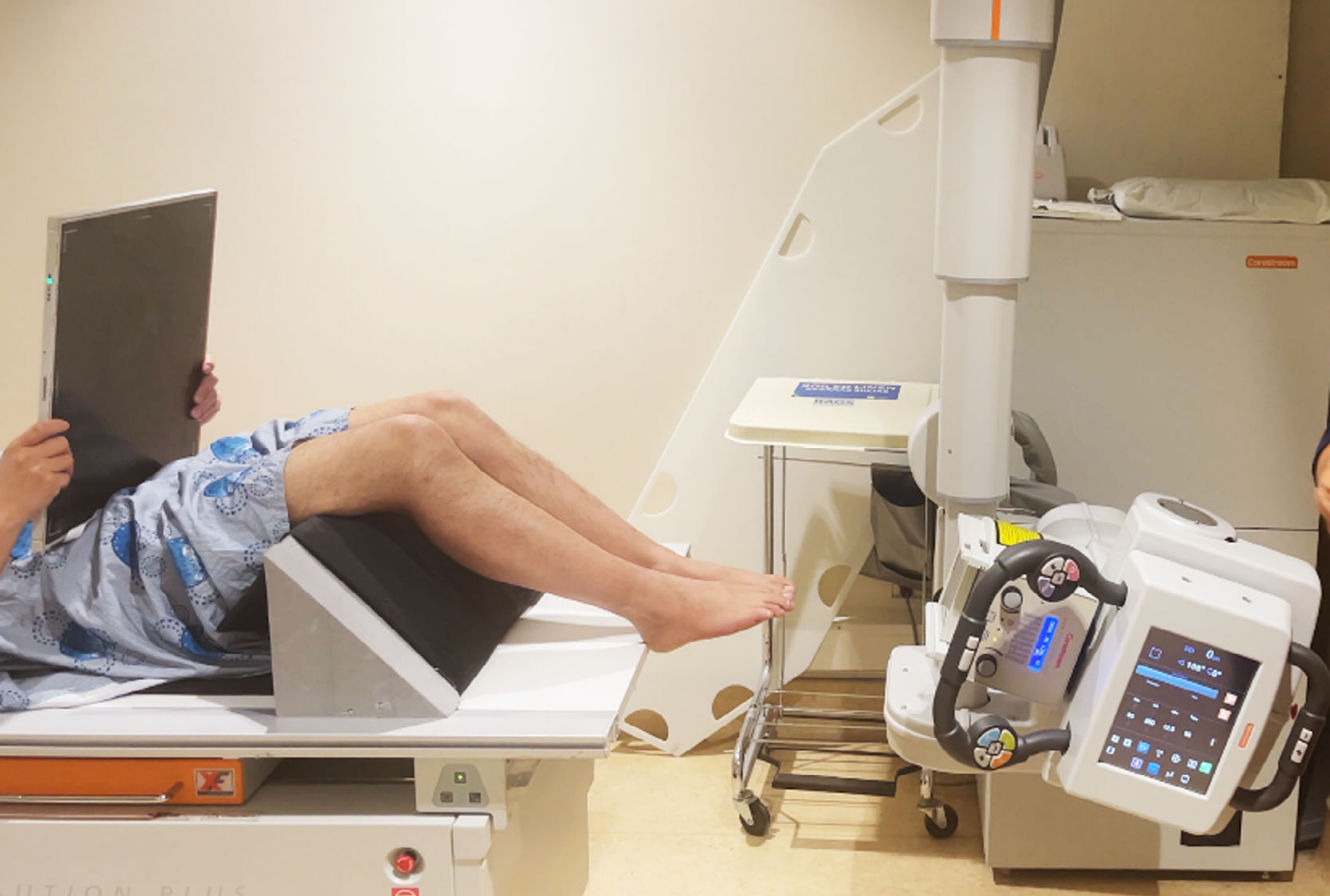
Clinical photograph showing positioning of patient for tibial sag radiograph.

**FIGURE 2:**
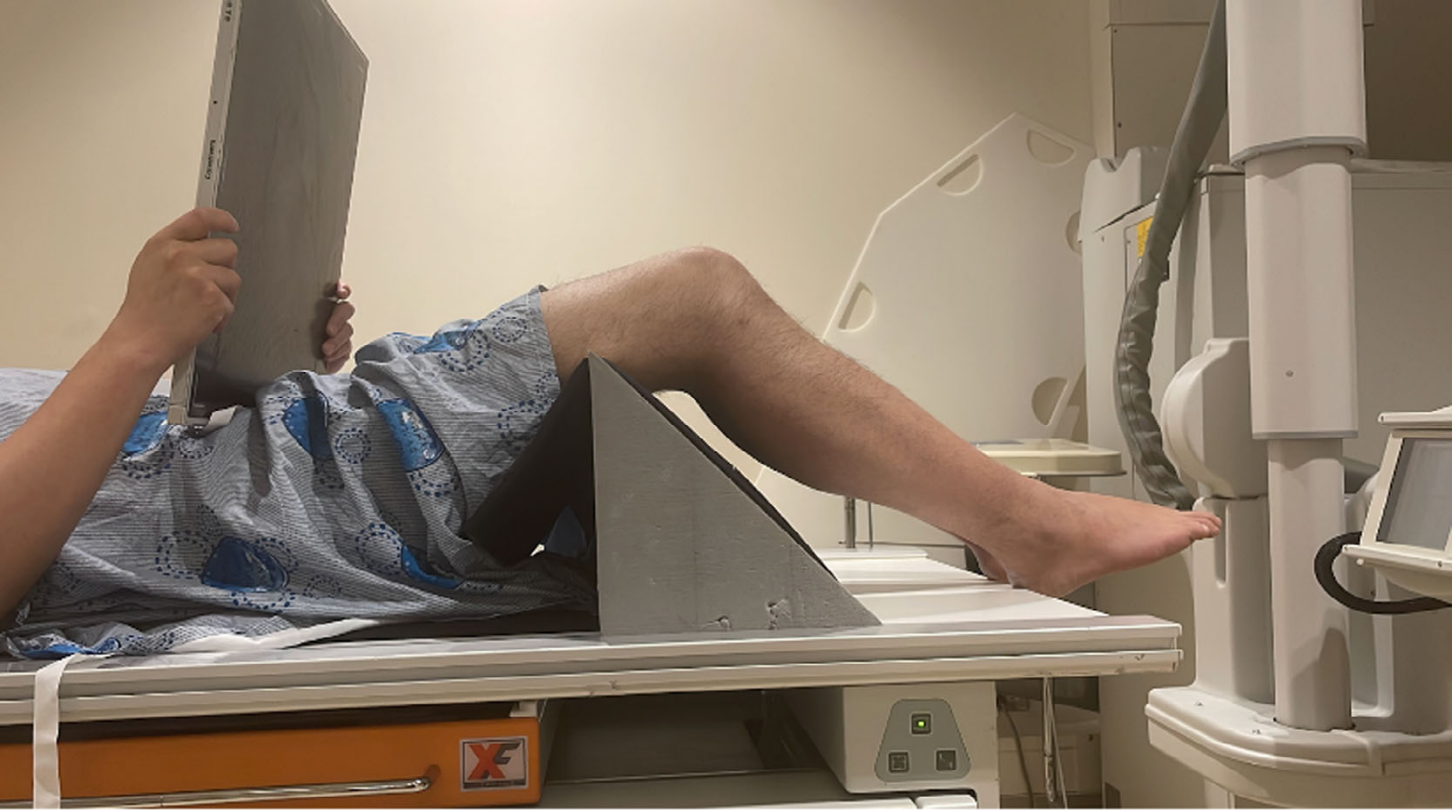
Clinical photograph showing positioning of radiolucent cushioned triangle. Note the parallel positioning of the cassette relative to the radiograph source.

The radiograph beam was directed from the patient's feet toward the knee, aiming 20° superior to horizontal. The radiograph beam was centered between both knees. The radiograph cassette was held by the patient, with the edge resting against their abdomen. The radiograph cassette was positioned 1.5 m from the radiograph source by the radiographic technician to standardize image magnification. The cassette was held parallel to the radiograph source (aiming 20° inferior).

The position of the tibia relative to the femur on tibial sag radiographs was measured in a standardized fashion. First, a tangential line was drawn across the most anterior aspect of the femoral condyles. A parallel line was then drawn at the most anterior aspect of the tibial plateau. The perpendicular distance between these lines represented the position of the tibia relative to the femur. The magnitude of tibial posterior sag was calculated by taking the difference in measurements between the injured and noninjured knees (Figure 3). A positive SSD was indicative of posterior displacement of the tibia on the injured side relative to the noninjured side.

**FIGURE 3:**
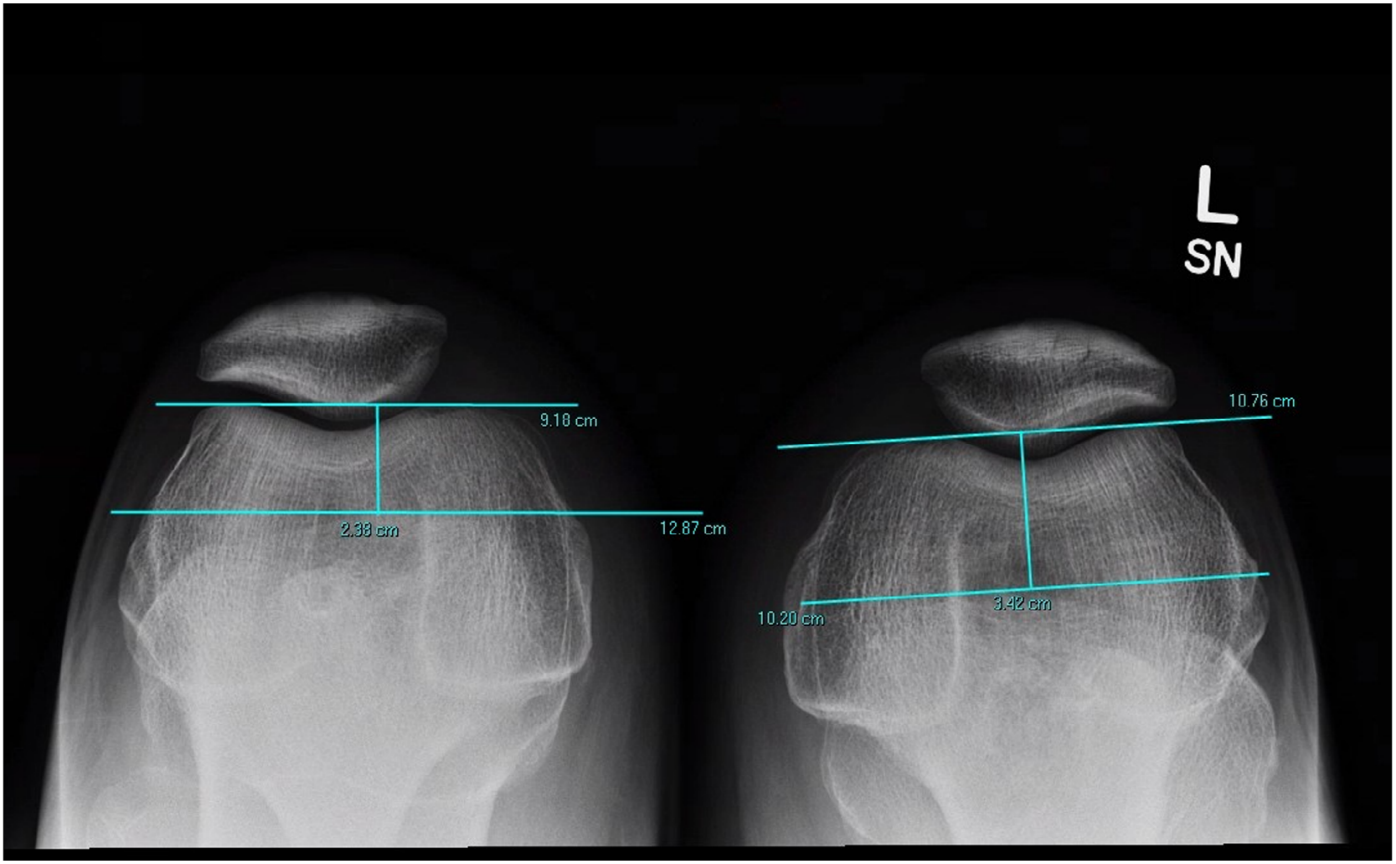
The tibial sag radiographic measurement technique. Sample tibial sag PCL radiographic images with corresponding measurements for a patient who presented with left knee grade III PCL laxity on clinical examination. The position of the tibia relative to the femur on the tibial sag radiograph was 23.8 mm on the right (uninjured) knee and 34.2 mm on the left (injured) knee. The resulting side‐to‐side difference was 10.4 mm, which indicates the relative posterior displacement of the tibia on the injured side in response to gravity stress. (PCL, posterior cruciate ligament.)

### Telos PCL Stress Radiograph Technique

Lateral Telos stress radiographs were obtained with the patient in a lateral decubitus position in 90° of knee flexion.^5^ A posteriorly directed force of 150 N was applied to the anterior aspect of the tibia, just distal to the tibial tuberosity. Comparative Telos stress radiographs were obtained from the uninjured and the injured knee. The posterior displacement of the tibia relative to the femur was calculated using methods described by Shultz et al. (Figure 4).^5^ SSD for posterior displacement in mm was calculated for each patient from both tibial sag and Telos stress radiographs.

**FIGURE 4:**
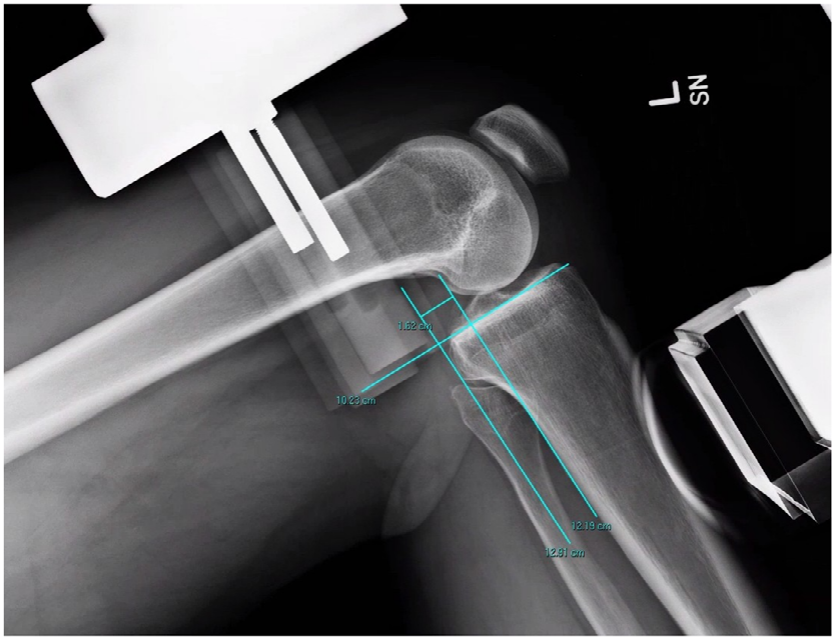
Telos stress radiograph measurement technique.

### Reliability Testing

Three senior orthopaedic trainees (G.W.S., D.L.L., C.C.) independently reviewed all corresponding tibial sag and Telos posterior stress radiographs. Each reviewer completed a standardized training session during which they showed proficiency using each measurement method. The reviewers were blinded to clinical laxity grades. All reviewers repeated tibial sag radiograph measurements for all radiographs to obtain data for the evaluation of intraobserver reliability, after a minimum washout period of 1 week to reduce recall bias. The sequence of radiographs was randomized, using a computer‐generated random sequence, to further reduce recall bias.

### Statistical Analysis

Baseline characteristics for the cohort were calculated using means and standard deviations and frequencies and proportions as appropriate. Summary statistics for each observer's radiograph measurements were calculated using means and standard deviations. Intraclass correlation coefficients (ICCs) with 95% confidence intervals (95% CIs) were calculated to assess the interobserver reliability of each stress radiography method using the Shrout and Fleiss Convention.^14^ We assumed our observers were randomly selected from a larger population of observers. In practice, a single clinician evaluates stress radiographs during patient assessments. Therefore, we calculated ICC (2,1) based on a single‐rating, absolute agreement, 2‐way random effects model.

ICC values were interpreted according to guidelines from Koo and Li.^15^ Values <0.5 indicated poor reliability, values between 0.5 and 0.75 indicated moderate reliability, values between 0.75 and 0.9 indicated good reliability, and values >0.9 indicated excellent reliability. The upper and lower bounds of the 95% CI for a given ICC point estimate were used to quantify the degree of reliability.^15^ For example, if the 95% CI for an ICC point estimate ranged from 0.65 to 0.80, then the reliability was considered moderate to good. Separate interobserver reliability ICC estimates were calculated for measurements on all tibial sag and Telos radiographs (injured and noninjured knees representing a unique observation) as well as the SSD measurements. We expected relatively weaker estimates of reliability for the SSD given that 2 radiographs are measured to derive SSD.

Intraobserver reliability estimates of tibial sag radiograph measurements were calculated based on a single rating, absolute agreement, and a 2‐way mixed effects model. ICC point estimates and 95% CIs were reported for each reviewer separately. All statistical tests were conducted using R Statistical Software (Vienna, Austria).

## RESULTS

Of the 108 patients with PCL injuries who presented during the study period, 74 patients underwent both tibial sag and Telos stress radiographs and were included in this study. Of these, 54 (73%) were men, and the mean age of the cohort was 34.4 ± 12.1 years (range, 14‐67 years). There were 26 (35.1%) grade I injuries, 39 (52.7%) grade II injuries, and 9 (12.2%) grade III injuries (Table 1). The mean SSD measured by the 3 reviewers ranged from 6.9 ± 4.2 mm to 7.2 ± 4.3 mm when using the tibial sag radiograph method and from 8.0 ± 4.0 mm to 8.4 ± 4.1 mm when using the Telos method (Table 2).

**TABLE 1:** Baseline Demographic for the Entire Cohort

**Characteristic**	**Value**
Age, mean (SD)	34.4 (±12.1) yr
Men, n (%)	54 (73%)
Multiligamentous knee injury, n (%)	46 (62.2%)
Clinical laxity grade, n (%)	
I	26 (35.1%)
II	39 (52.7%)
III	9 (12.2%)
Laterality, n (%)	
Left	41 (55.4%)
Right	33 (44.6%)

SD, standard deviation; yr, year.

**TABLE 2:** Descriptive Data for Side‐To‐Side Differences Recorded by All Reviewers

	**Tibial Sag**	**Telos**
Reviewer 1	7.2 ± 4.3	8.4 ± 4.1
Reviewer 2	7.1 ± 4.3	8.3 ± 4.8
Reviewer 3	6.9 ± 4.2	8.0 ± 4.0

*Note*: Data reported as mean distance in mm ± standard deviation.

The ICC (2,1) for measuring the position of the tibia relative to the femur on all radiographs was 0.95 (95% CI 0.93‐0.96) for the tibial sag method, indicating excellent interobserver reliability, whereas the ICC (2,1) for the Telos method was 0.83 (95% CI 0.79‐0.87), indicating good interobserver reliability. The ICC (2,1) for measuring SSD using the tibial sag method was 0.89 (95% CI 0.84‐0.92), indicating good to excellent interobserver reliability, whereas the Telos method showed moderate to good interobserver reliability (ICC 0.70, 95% CI 0.59‐0.78) (Table 3). The intraobserver reliability of measuring SSD using the tibial sag method was excellent for 2 reviewers (ICC 0.96, 95% CI 0.94‐0.97; ICC 0.95, 95% CI 0.92‐0.97) and good to excellent for 1 reviewer (ICC 0.91, 95% CI 0.86‐0.94).

**TABLE 3:** Interobserver Reliability of Tibial Sag and Telos Stress Radiography

**Parameter**	**Tibial Sag**	**Telos**
All radiographs, mm	0.95 (0.93‐0.96)	0.83 (0.78‐0.87)
Side‐to‐side difference, mm	0.89 (0.84‐0.92)	0.70 (0.59‐0.78)

*Note*: Data reported as intraclass correlation coefficients (2,1) (95% confidence intervals).

## DISCUSSION

In this study, we found evidence of acceptable intraobserver and interobserver reliability when using tibial sag knee radiographs for the evaluation of patients with PCL injuries.

The tibial sag radiograph method leverages the clear anatomic landmarks of the anterior femoral condyles and anterior aspect of the tibia, which can be easily identified by the radiograph reviewer. Particularly, in young patients, these anatomic areas are generally devoid of osteophytic changes, which facilitates accurate landmark identification. Moreover, the tibial sag technique only requires 2 landmarks to be identified with 3 corresponding measurement lines, versus 3 landmarks and 4 corresponding measurement lines for the Telos method, which inherently reduces opportunities for measurement error. Telos measurements are widely used within the literature in PCL injury studies, as the Telos device produces an adjustable, quantifiable, and reproducible posterior force on the injured knee.^2^, ^8^ Our findings of comparatively lower interobserver reliability of the Telos method may be because of multiple factors. The Telos method requires consistent identification of the posterior tibial slope and the posterior aspect of the femoral and tibial condyles. Identification of these radiographic landmarks can be subjective and is reliant on acquisition of a high‐quality lateral knee radiograph. Imperfect lateral radiographs without adequate overlap of the respective medial and lateral femoral and/or tibial condyles can create ambiguity during radiograph measurement, which may explain the lower reliability of the Telos method in our study.

The findings from our study support the use of the tibial sag radiography method as a reliable alternative to other forms of stress radiography such as Telos and kneeling methods. The tibial sag method has advantageous properties when compared with these other radiographic methods. Ligamentous stress from tibial sag radiographs is obtained by patient positioning and gravity, which may more closely replicate the loads experienced during daily activities when compared with the Telos method, as the induced stress is proportional to the weight of a patient's limb. As well, the test is more tolerable for those with anterior knee pain. Prior evidence has shown significantly higher pain scores during Telos and kneeling stress radiographs when compared with gravity stress radiographs, resulting in a large proportion of patients (88%) preferring gravity methods.^3^, ^9^ Moreover, the setup and administration of tibial sag radiographs are relatively easy from a technical standpoint. Previous research has shown faster radiograph acquisition times when performing gravity stress radiography versus Telos and kneeling methods.^3^ Finally, no additional equipment is required, which may be advantageous in low‐resource environments. These advantageous properties make the gravity stress radiograph technique appealing, particularly when used to quantify laxity for the purpose of clinical research.

### Limitations

This study is not without limitations. First, the sample size was small, especially the low number of grade III PCL injuries. Next, it is possible for the anterior tibial border to overlap with the distal femur, which may introduce ambiguity when measuring tibial sag radiographs. Because of the low number of women included in our study, we were unable to disaggregate the data by sex. Additionally, our institution has familiarity with this radiographic technique; therefore, it is possible that there may be a learning curve in acquiring radiographs when implementing the tibial sag technique at other institutions. Next, the washout period for the assessment of intraobserver reliability was relatively short. However, given the relatively high volume of radiographs measured and randomization of the sequence of radiographs, it is unlikely that observers were affected by recall bias. Multiligamentous knee injuries can impact the rotation of the knee, leading to an overestimation or underestimation of the anterior aspect of the tibia. This was not directly explored in this study, but by ensuring that the patella and knees remained centered during the radiograph acquisition, the tibial sag radiograph method minimizes any concern with tibial rotation. Further evidence is required to determine whether this technique provides a valid estimate of PCL laxity.

## CONCLUSIONS

Tibial sag radiography is a reliable method that can be used to identify posterior displacement of the tibia relative to the femur as measured by SSD in patients with PCL injuries.

## DISCLOSURES

The authors (G.W.S., D.L.L., C.C., G.H., D.B.W.) declare that they have no known competing financial interests or personal relationships that could have appeared to influence the work reported in this article.
